# Repeated cross-sectional sampling of pigs at slaughter indicates varying age of hepatitis E virus infection within and between pig farms

**DOI:** 10.1186/s13567-022-01068-3

**Published:** 2022-07-07

**Authors:** Marina Meester, Martijn Bouwknegt, Renate Hakze-van der Honing, Hans Vernooij, Manon Houben, Sophie van Oort, Wim H. M. van der Poel, Arjan Stegeman, Tijs Tobias

**Affiliations:** 1 Department of Population Health Sciences, Faculty of Veterinary Medicine, Farm Animal Health Unit, Utrecht University, Yalelaan 7, 3584CL Utrecht, the Netherlands; 2 Vion Food Group, Boseind 10, 5281 RM Boxtel, the Netherlands; 3 Wageningen Bioveterinary Research, Houtribweg 39, 8221 RA Lelystad, the Netherlands; 4 Royal GD, Arnsbergstraat 7, 7418 EZ Deventer, the Netherlands

**Keywords:** HEV, virus, zoonosis, population infection dynamics, seroprevalence, within-farm transmission, batch sampling

## Abstract

Humans can become infected with hepatitis E virus (HEV) by consumption of undercooked pork. To reduce the burden of HEV in humans, mitigation on pig farms is needed. HEV is found on most pig farms globally, yet within-farm seroprevalence estimates vary considerably. Understanding of the underlying variation in infection dynamics within and between farms currently lacks. Therefore, we investigated HEV infection dynamics by sampling 1711 batches of slaughter pigs from 208 Dutch farms over an 8-month period. Four farm types, conventional, organic, and two types with strict focus on biosecurity, were included. Sera were tested individually with an anti-HEV antibody ELISA and pooled per batch with PCR. All farms delivered seropositive pigs to slaughter, yet batches (resembling farm compartments) had varying results. By combining PCR and ELISA results, infection moment and extent per batch could be classified as low transmission, early, intermediate or late. Cluster analysis of batch infection moments per farm resulted in four clusters with distinct infection patterns. Cluster 1 farms delivered almost exclusively PCR negative, ELISA positive batches to slaughter (PCR^−^ELISA^+^), indicating relatively early age of HEV infection. Cluster 2 and 3 farms delivered 0.3 and 0.7 of batches with intermediate infection moment (PCR^+^ELISA^+^) respectively and only few batches with early infection. Cluster 4 farms delivered low transmission (PCR^−^ELISA^−^) and late infection (PCR^+^ELISA^−^) batches, demonstrating that those farms can prevent or delay HEV transmission to farm compartments. Farm type partly coincided with cluster assignment, indicating that biosecurity and management are related to age of HEV infection.

**Supplementary Information:**

The online version contains supplementary material available at 10.1186/s13567-022-01068-3.

## Introduction

Hepatitis E virus (HEV) genotype 3 and 4 are zoonotic viruses with pigs as a main reservoir. In pigs HEV infections normally run an asymptomatic course. In humans HEV infection is often asymptomatic as well, yet can be life-threatening in risk populations [1, 2]. Humans can become infected by pigs via direct and indirect contact or the consumption of contaminated raw or undercooked pork [3–5]. In order to reduce the exposure of humans to the virus, there is a need to reduce the number of HEV infected slaughter pigs [6].

HEV is endemic in pig farms worldwide and nearly all farms are affected (farm-level seroprevalence often reported close to 100%), regardless of the country of origin of the pigs [7]. Yet the within-farm prevalences of HEV and thus the underlying infection dynamics vary considerably [8, 9]. Understanding variation in dynamics within and between farms will provide knowledge on how to prevent transmission of HEV within farms. Cohort studies have given insight in the general course of HEV infection in pig farms. Summarized, pigs have maternal antibodies during the first 6–9 weeks of age, that protect against infection during the farrowing phase. Shedding often starts at the end of the nursery phase, with a peak in number of shedders a few weeks after the start of the fattening phase. Most fattening pigs have antibodies against HEV and no longer shed the virus at time of slaughter [10–13]. Cohort studies are common to study infection dynamics, but as these are time-consuming, expensive and require sampling a large number of live animals, and consequently an ethical justification, often only few batches on a farm can be studied simultaneously. Hence, to gain insight into variation between batches and farms it is desirable to carry out a large scale study of HEV population dynamics of infection in a different manner.

By using blood samples collected from multiple batches of slaughter pigs, for both detection of HEV RNA (PCR) and antibodies (ELISA), classification of the infection status at batch level is possible. A slaughter batch is defined as all pigs slaughtered on the same day and originating from one unique farm. The status of infection at batch level can be classified as “low transmission” when results for both PCR and ELISA are negative, “early” when pigs test positive for antibodies and negative in PCR, “intermediate” when positive for both tests and “late” when pigs test negative for antibodies but positive in PCR. By this approach of batch classification, it may be possible to identify at approximately what age we should intervene to reduce the proportion of HEV infected slaughter pigs and whether this differs between farms or even within farms, between farm compartments.

Farm type may be associated with population dynamics of HEV infections, as was shown for organic farms that are known for high seroprevalences [14]. For other farm types it is not clear whether these are related to HEV population infection dynamics, but farm biosecurity has been suggested as essential in reducing HEV [8, 15]. Here, the aim is to investigate whether farms, of different farm types, cluster based on patterns in batch level HEV infection dynamics, and whether that can be used to determine the relative age of infection on farms.

## Materials and methods

### Study design and sample size calculations

A repeated cross-sectional study was performed on batches of finishing pigs at slaughter, from farms in the Netherlands. Sampling took place between January and August 2019 at three abattoirs of one slaughter company. To determine farm sample size, calculations to estimate the true prevalence were made. Based on a previous seroprevalence study in the Netherlands [14], we assumed an 80% within-farm seroprevalence in high prevalence farms and a 20% within farm-seroprevalence in low prevalence farms. An error rate of 15% and 95% confidence and modest test sensitivity and specificity of 80%, returned 208 farms to be sampled [16]. To allow for loss to follow-up of farms (i.e. farms that stop delivering to the slaughter company), additional farms were included in this study, resulting in the sampling of 215 farms. To determine within-farm sample size, calculations to estimate a simple proportion of infected pigs were made, assuming 80% seropositive pigs per farm, a desired precision of 0.15, a 95% confidence interval and a population size of 1000 fattening pigs, returning 28 required samples per farm. The same sample size was obtained similarly for farms with 20% seropositive pigs [17].

### Farm selection

In previous studies, farm type was related to the seroprevalence of HEV on farms [14, 18]. To ensure inclusion of specific farm types that may be underrepresented in random sampling, four distinct types of farms were included. These farm types are defined as follows: (A) Organic farms: Farms that produce pigs organically according to the European Commission Regulation (EC889/2008), including the obligation for outdoor access for pigs. (B) HyCare farms: Farms that were listed to have an assumed better than average internal biosecurity, by working according to the HyCare^®^ concept [19]. The HyCare concept includes non-porous and easy to clean coating on floors and walls; compliance to cleaning and disinfection procedures targeted at viruses and bacteria commonly present in most pig farms; preventive pest control; applying standard treatment of pig drinking water and a working method focused on prevention of spread of infectious agents. (C) High health farms: Farms with an assumed better than average external biosecurity, because either farms are farrow-to-finish farms that are classified by the owner as being specific pathogen free (SPF) or fattening-to-finish or weaning-to-finish farms that purchase their pigs from SPF farms or farrow-to-finish farms that may acquire animals once every 6 weeks from only one farm that has a higher health status. (D) Conventional farms: All farms that rear finishing pigs that do not fit the definition of organic, HyCare or high health farms.

In order to collect at least 28 samples per farm, it was arbitrarily decided to collect six samples per batch, from at least five batches per farm. To enable sampling of five batches per farm within the time-frame of the study, an inclusion criterion for conventional farms was that they delivered five or more batches to the slaughter company per 6 months in the preceding year 2018. After applying that inclusion criterion, 897 of 2493 farms were eligible for this study, of which 175 conventional farms were randomly selected with the randomize tool in MS Excel [20]. These 175 farms delivered on average seven batches of slaughter pigs per 6 months in 2018. The criterion was also applied to randomly select 20 organic farms from 55 eligible farms (of a total of 74), delivering on average five batches per 6 months in 2018. As the number of eligible HyCare and high health farms is limited, all farms that met the given definitions and the inclusion criterion of delivering sufficient batches were included in the study, resulting in ten HyCare and 20 high health farms with on average eight and 22 batches per 6 months delivered in 2018.

### Sample collection

From every farm included in the study, at least five slaughter batches delivered to the abattoir were sampled. Per slaughter batch, six pigs were randomly selected and whole blood samples were collected during exsanguination. Treated test tubes (10 mL) for serum collection with coagulation inducer were used. Until coagulation, samples were stored at room temperature and after centrifugation at 1000 *g*, stored at −20 °C until analysis.

### Detection of anti-HEV antibodies

Individual sera were tested for the presence of IgM and IgG antibodies against HEV using an in-house pig specific sandwich enzyme-linked immunosorbent assay (ELISA). The development of the immunoassay has been described in detail before [21]. In short, the ELISA was developed using a recombinant Baculovirus expression product of HEV ORF-2, genotype 3 and coated onto polystyrene ELISA plates. Bound HEV antibodies are detected by anti-porcine IgG and IgM antibodies labelled with horse radish peroxidase (HRPO) and visualized by incubation with a ready-to-use 3,3',5,5'-Tetramethylbenzidine (TMB) substrate. The optical density (OD) of the substrate was measured by an ELISA plate reader at 450 nm and compared to positive control samples. The estimated diagnostic sensitivity and specificity of the ELISA are 84% and 89%, respectively [21]. The first two third of collected sera was tested with ELISA plates with the original coated antigen described by van der Poel et al. [cut-off at 24.5 Percentage Positive (PP)] [21], after which plates ran out. The remaining one third was tested with the same ELISA, but with a different procedure to purify antigen and a lower coating dilution. Possible bias due to this change was examined, showing that within-farm proportions of positive ELISA samples were not significantly different (Additional file 1).

### Detection of HEV RNA

For the molecular detection of HEV RNA, pig serum was pooled per slaughter batch to a total of 200 µL serum. The volume of individual sera per pool therefore depended on the number of samples obtained from each batch, but was usually 33.3 µL. The pools were mixed to a final dilution of 1–3 in 600 µL TRIzol LS (Invitrogen, Sanbio, Uden, Netherlands), added to maintain RNA for subsequent HEV RNA detection, and mixed thoroughly. After five minutes of incubation at room temperature the samples were stored at −20 °C before further analysis. Before starting RNA isolation, the TRIzol mixture was centrifuged in a tabletop centrifuge for one minute at 13 000 *g*. Four hundred µL of the TRIzol mixture was used to extract RNA with the Direct-zol 96 kit (Zymo Research, Irvine, CA, USA). RNA was used immediately for HEV RT-PCR or stored at −70 °C until further testing. HEV detection by real-time RT-PCR was performed on undiluted RNA samples with primers JVHEVF and JVHEVR [22] with the Taqman Fast virus-1 step master mix (Applied biosystems, Waltham, MA, USA). Ct-values lower than 40.0 were classed positive.

### Statistical analysis

The descriptive and analytic statistics were performed using R software version 4.0.3 [23], with the packages readxl [24], dplyr [25], tidyverse [26], lme4 [27], cluster [28], ggplot2 [29], factoextra [30] and nbClust [31]. Overall mean within-farm seropositive proportion was calculated by dividing the number of ELISA positive sera by the total number of sera collected per farm. Mean within-farm proportion of PCR positive batches was calculated by dividing the number of PCR positive pools by the number of PCR pools (i.e. batches of pigs) sampled per farm. The aggregated results per farm were used to summarize the data per farm type by calculating mean, median and interquartile range (IQR: 25^th^ to the 75^th^ percentile).

Mixed-effect logistic regression modelling was used to assess the association between the ELISA or PCR results and farm type, and the association between ELISA and PCR results on batch level, with PCR outcome as dependent variable. To account for dependence among test results due to repeated measurements within batches nested within farms in course of time, the random effects part of the models was determined first. Different random effects were added, to a model that included all fixed effects. The tested random effects, for both ELISA and PCR result as outcome, are given in the first part of Table 1. The model with lowest Akaike’s Information Criterium (AIC) of all competing models was selected as this is assumed to contain the best random effects for analysing outcome variable ELISA and PCR respectively [32]. After that, the fixed effects were determined by backward model selection based on model fit with AIC.

**Table 1:** **Tested random and fixed effects in mixed effect logistic regression models with ELISA result and PCR result as outcome variables**.

Random effects of mixed effect logistic regression models (with all fixed effects included)		
Outcome	Variable name	Explanation
ELISA result (0/1 per pig)	1 | farm	Random intercept per farm
	month–1 | farm	Random slope per farm
	month | farm	Random intercept and slope per farm
	(1 | farm / batch)	Random intercept per batch, nested in farm
PCR result (0/1 per batch)	1 | farm	Random intercept per farm
	month–1 | farm	Random slope per farm
	month | farm	Random intercept and slope per farm
Fixed effects (independent variables) of mixed effect logistic regression models		
ELISA result (0/1 per pig)	farm type	Conventional; organic; high health; HyCare
	ELISAtest	Original; alternative
PCR result (0/1 per batch)	farm type	Conventional; organic; high health; HyCare
	batch seropositive proportion	Between 0 and 1 per batch

0 = negative, 1 = positive test result

For the ELISA results as outcome, the full model contained farm type and ELISA test (newly coated vs. original) as fixed effects. The full model with PCR results as outcome contained farm type and batch proportion of seropositive samples as fixed effects (Table 1, fixed effects). Results of the models are presented as odds ratios (OR) with 95% confidence intervals (CI).

Patterns in infection status of batches within and between farms were investigated via k-means clustering. K-means clustering is an unsupervised learning algorithm, that allocates observations to clusters, by minimizing distances between observations within clusters (i.e. the lowest within-cluster variation) and maximizing distances between clusters (i.e. the highest between-cluster variation) [33]. Six steps were taken in this analysis.

First, the ELISA positive proportion per batch was dichotomized, with zero or one out of six samples positive being defined as “ELISA negative” (ELISA^−^) and two or more out of six samples positive defined as “ELISA positive” (ELISA^+^). Second, each batch was assigned to one of four classes, based on the combined ELISA en PCR test results. The batch classes were designed to represent the relative age of HEV infection of fattening pigs in a farm compartment: batches without or with only one HEV infected pig (PCR^−^ELISA^−^, “low transmission”), batches with pigs being HEV infected just before slaughter and without antibodies developed (PCR^+^ELISA^−^, “late”), batches with pigs being HEV infected longer ago, with pigs being viraemic at slaughter and antibodies having developed in two or more pigs (PCR^+^ELISA^+^, “intermediate”) and batches with infections that occurred a long time ago, with no viraemic pigs within the batch while antibodies have developed in two or more pigs (PCR^−^ELISA^+^, “early”). The number of batches for each category was divided by the total number of sampled batches per farm to obtain batch proportions for the four categories. Three out of four proportions were used in the k-means algorithm (PCR^−^ELISA^+^, PCR^+^ELISA^+^, PCR^+^ELISA^−^), as the fourth one is redundant because the proportions add up to one. Third, the number of clusters (k) was determined by NbClust, a function that provides a variety of methods (of which 23 functioned for the current proportion data) to find the optimal number of clusters [31]. Fourth, k-means clustering was performed, using k randomly selected initial cluster centres and assignment of observations (farms) to the closest centres based on the Euclidean distance between the farm and the centre. After assignment of all farms to a cluster, the cluster centre was updated by calculating new mean values of the three batch proportions per cluster. Based on the updated cluster centres, assignment of farms to clusters was repeated and these steps were iterated until the cluster assignment stopped changing [34]. As the result of k-means clustering is sensitive to the random starting cluster centres, 50 different starting assignments were addressed, and the algorithm selects the results corresponding to the starting assignment with the lowest within cluster variation. To evaluate the performance of clustering, the within cluster sum of squares per cluster and the proportion of between sum of squares to total sum of squares were calculated.

## Results

### Exclusion from analysis

In total 11 115 individual blood samples, obtained from 1930 slaughter batches, were collected. Six hundred samples were excluded from lab or data analysis for several reasons: 25 samples were excluded because the tubes broke during processing; another five were excluded because the tubes showed to be empty when arriving at the lab; batches with less than five or more than 12 samples were excluded from the data analysis (468 samples, 203 batches); lastly, farms with less than four batches were excluded from data analysis (102 samples, 16 batches, seven farms). This resulted in data of 208 farms, with a total of 1711 batches and 10 515 samples.

### ELISA and PCR results at farm-level and within-farms

**Figure 1:**
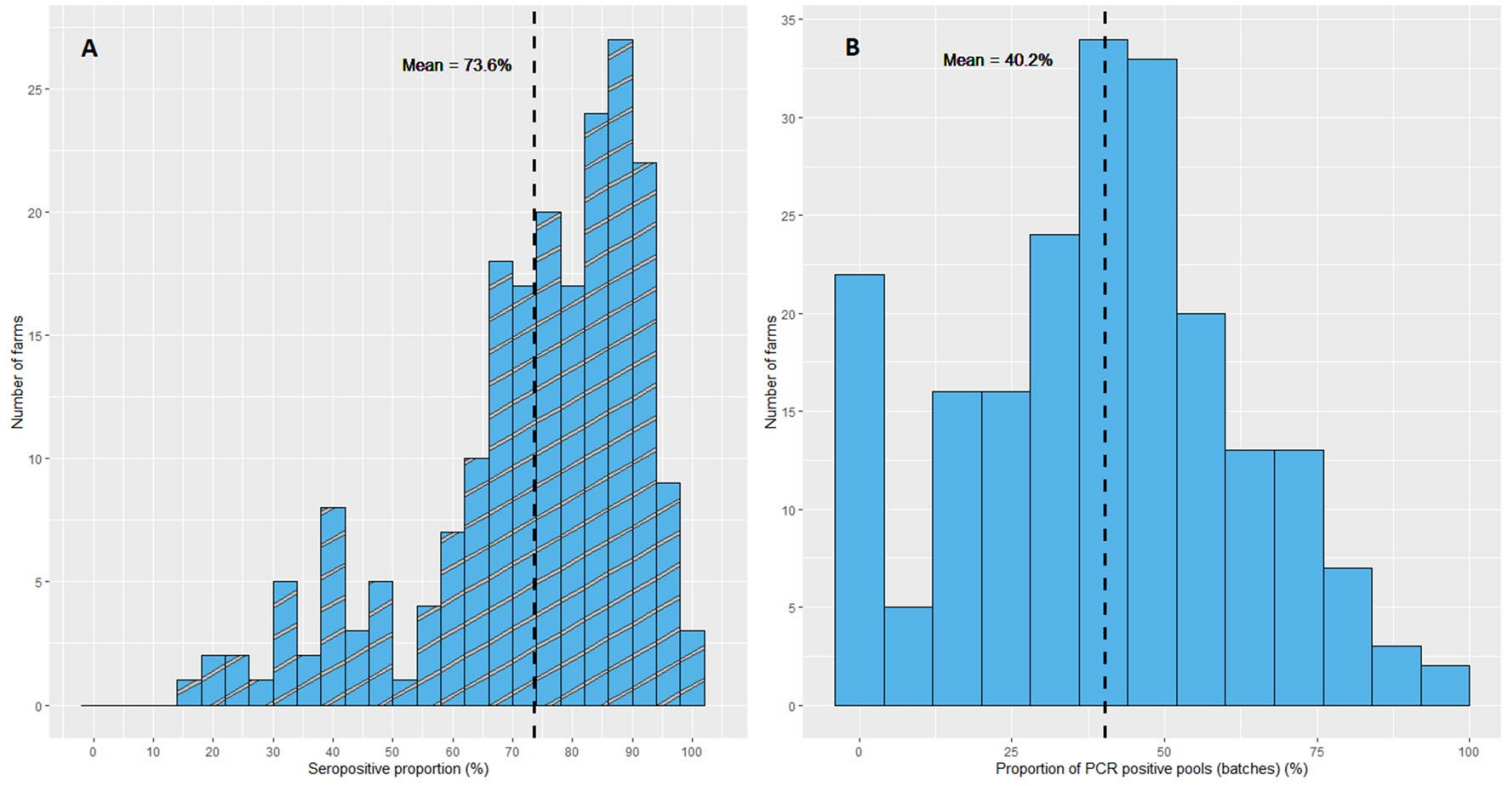
**Histogram of within-farm seropositive and batch PCR positive proportions.** **A** Antibody ELISA positive proportions within farms; **B** HEV RNA PCR positive pool (batch) proportions within farms.

All farms delivered at least one seropositive pig to slaughter. The mean within-farm seropositive proportion of slaughter pigs was 73.6% (IQR 66.7–87.2%). The lowest within-farm seropositive proportion was 16.7% and 11 farms had a seropositive proportion above 95% (Figure 1A, Table 2). On average 40.2% of pools per farm tested HEV PCR positive, indicating at least one viraemic pig in these batches. It was estimated that this pool-level PCR positive percentage corresponds to an individual percentage of viraemic pigs per farm of 9.6% (95% CI 8.7–10.6%) (Additional file 2). Twenty-two farms (10.6%) delivered only HEV PCR negative batches to the abattoir (Figure 1B).

**Table 2:** **Baseline table of number of samples and batches, farm-level and within-farm results, for all farms and per farm type**.

Farm type Variable	All farms	Conventional farms	Organic farms	High health farms	HyCare farms
Number of samples	10 515	7202	941	1182	1185
Median number of samples per batch (IQR)	6 (6–6)	6 (6–6)	6 (6–6)	6 (6–6)	6 (6–6)
Number of batches	1711	1202	158	185	166
Number of farms	208	162	20	16	10
Median number of batches per farm (IQR)	8 (6–9)	8 (6–9)	8 (8–9)	13 (9–14)	18 (13–21)
Farm-level percentage with ≥ 1 seropositive pig at slaughter	100%	100%	100%	100%	100%
Mean within-farm seropositive percentage (IQR)	73.6% (66.7–87.2%)	73.8% (66.1–87.5%)	81.4% (76.6–87.2%)	70.2% (40.9–86.8%)	66.8% (60.9–75.5%)
Farm-level percentage ≥ 1 PCR positive batch at slaughter	89.4%	94.4%	15.0%	100%	100%
Mean within-farm percentages of PCR positive batches (IQR)	40.2% (25.0–57.1%)	44.0% (30.0–59.6%)	8.33% (0.00–12.5%)	41.0% (25.8–56.7%)	42.8% (38.8–47.0%)
Percentage of farms ≥ 1 PCR^−^ELISA^−^ batch	32%	29%	29%	44%	70%

### Farm types and mixed-effect logistic regression

**Figure 2:**
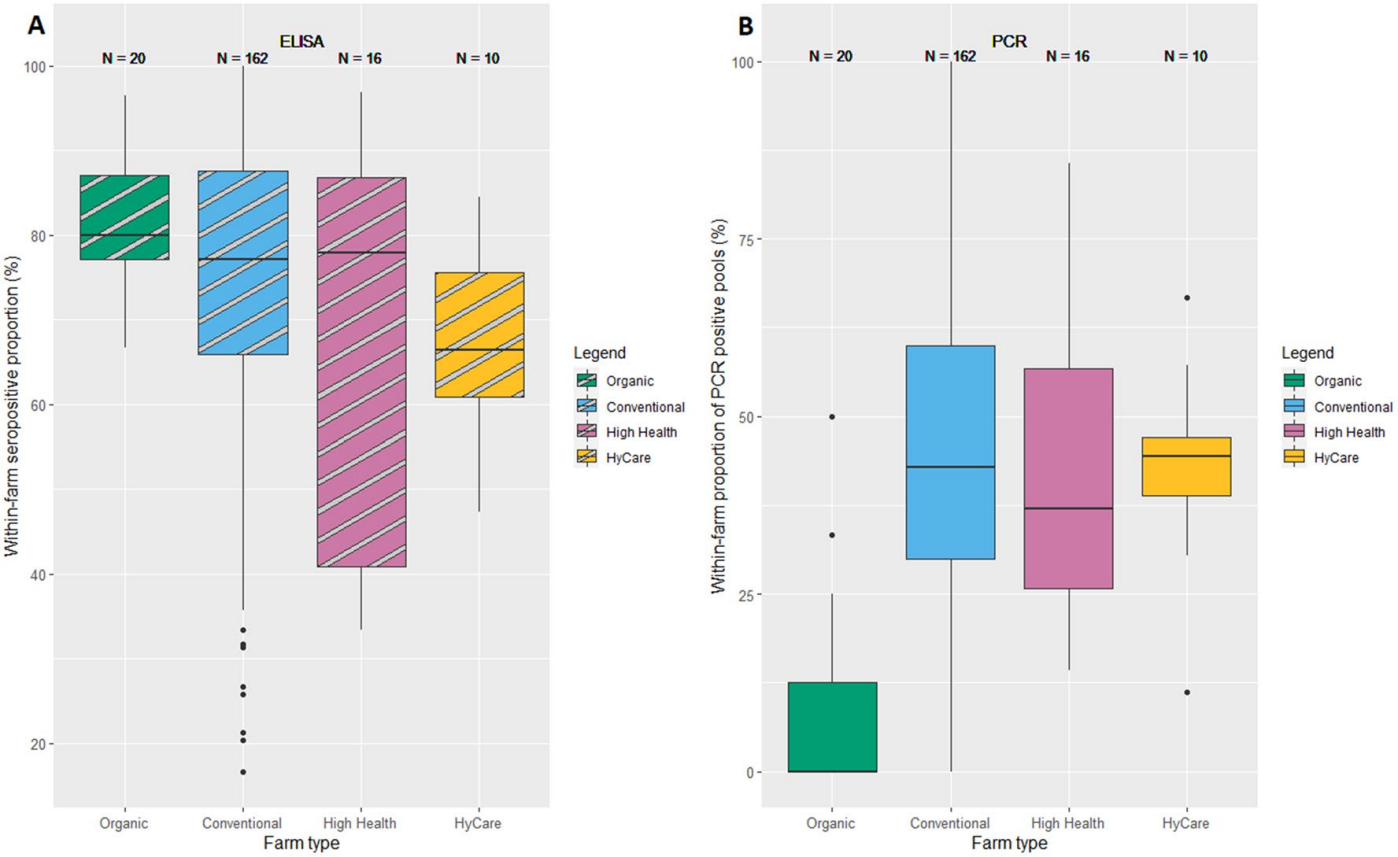
**Boxplots of within-farm seropositive and batch PCR positive proportions for four farm types.** Boxes represent the middle 50% of the values, marked with three horizontal lines that represent the first quartile (bottom), the median (middle) and third quartile (upper line). **A** Within-farm seropositive proportions per farm type; **B** Within-farm PCR positive pool (batch) proportions per farm type.

The within-farm ELISA positive proportions were comparable with regard to the median for conventional, organic and high health farms (Figure 2A). The results of high health farms are more skewed, i.e. there is a wider range in seropositive proportions below the median, compared to conventional and organic farms. The HyCare type had a lower median within-farm seropositive proportion of 66%. In the final logistic regression model, with a random intercept for batches nested within farms, both farm type and ELISAtest were dropped according to AIC. However, in the full model the OR for having an ELISA positive pig was lower for conventional, high health and HyCare farms opposed to organic farms, and for HyCare farms this difference was significant (Table 3). HyCare farms did not have a significantly lower OR than conventional or high health farms for the ELISA results.

**Table 3:** **Odds ratios with 95% confidence intervals of the full and final mixed effect logistic regression models for ELISA and PCR results as outcomes**.

ELISA results					
	*Full model*			*Best fitting model*	
Fixed effect	Odds ratio		95% CI	Odds ratio	95% CI
Farm type					
Organic	Ref.		Ref.	Dropped during backward selection by AIC	
Conventional	0.60		0.33–1.08		
High health	0.52		0.22–1.21		
HyCare	0.35		0.14–0.88*		
ELISA test					
Original	Ref.		Ref.	Dropped during backward selection by AIC	
Alternative	1.02		0.79–1.32		

PCR results				
	*Full model*		*Best fitting model*	
Fixed effect	Odds ratio	95% CI	Odds ratio	95% CI
Farm type				
Organic	Ref.	Ref.	Ref.	Ref.
Conventional	9.61	5.19–17.8*	9.69	5.24–17.9*
High health	7.54	3.58–15.9*	7.67	3.66–16.1*
HyCare	9.25	4.38–19.6*	9.39	4.45–19.8*
Batch seropositive proportion	0.88	0.62–1.24	Dropped during backward selection by AIC	

*the odds ratio is significantly different from the reference category.

The final logistic regression model with PCR result as outcome fitted best to the data with a model with random slope per farm and farm type as fixed effect. Conventional, high health and HyCare farms have an OR for having a PCR^+^ batch of 7.5–9.6 compared to organic farms (Table 3). The ORs of high health and HyCare types compared to conventional farms were not significantly different with regard to the PCR results (supported by Figure 2B).

### Clustering of farms by batch infection status

**Figure 3:**
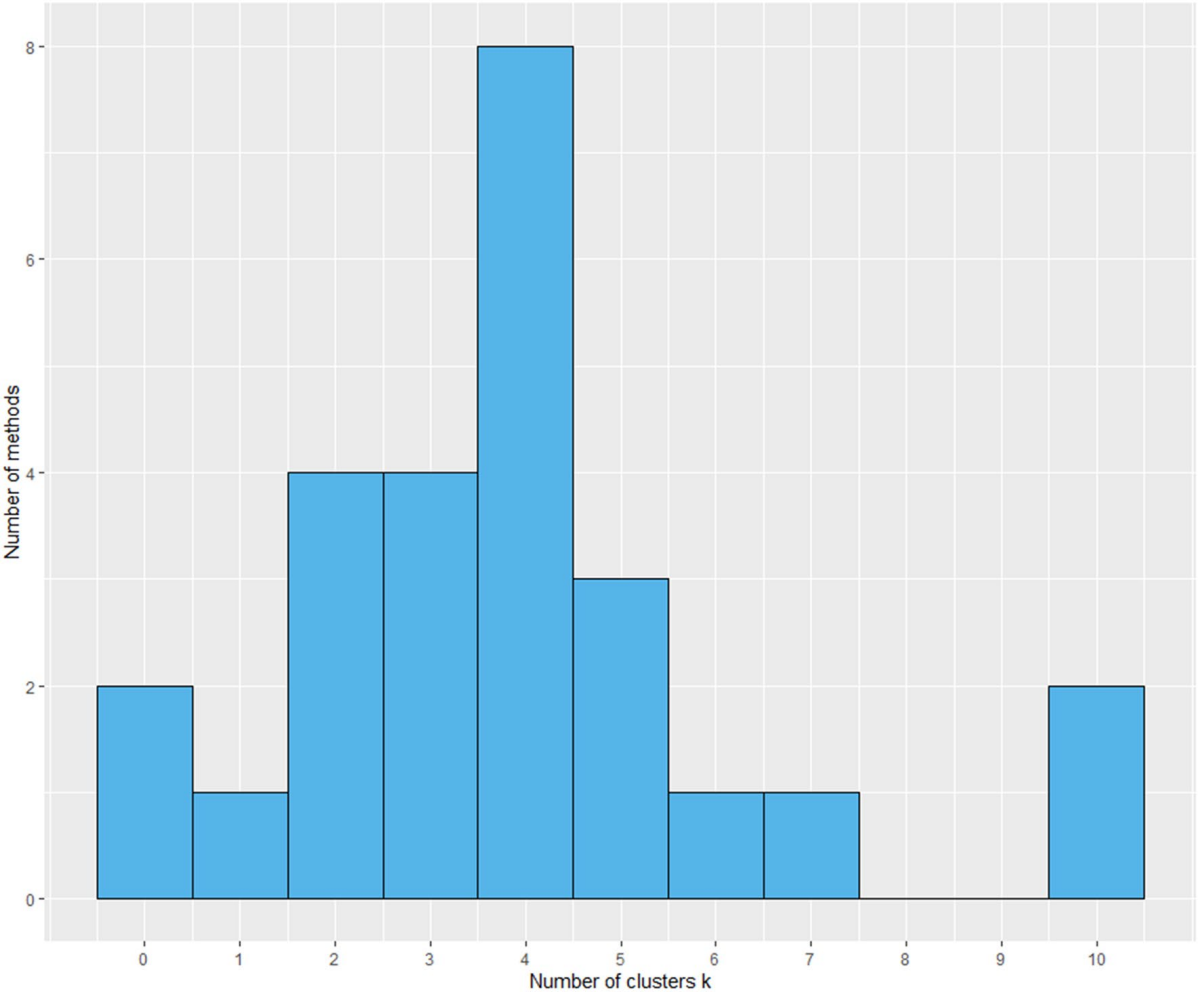
**Results of optimal number of clusters according to 23 methods**.

**Figure 4:**
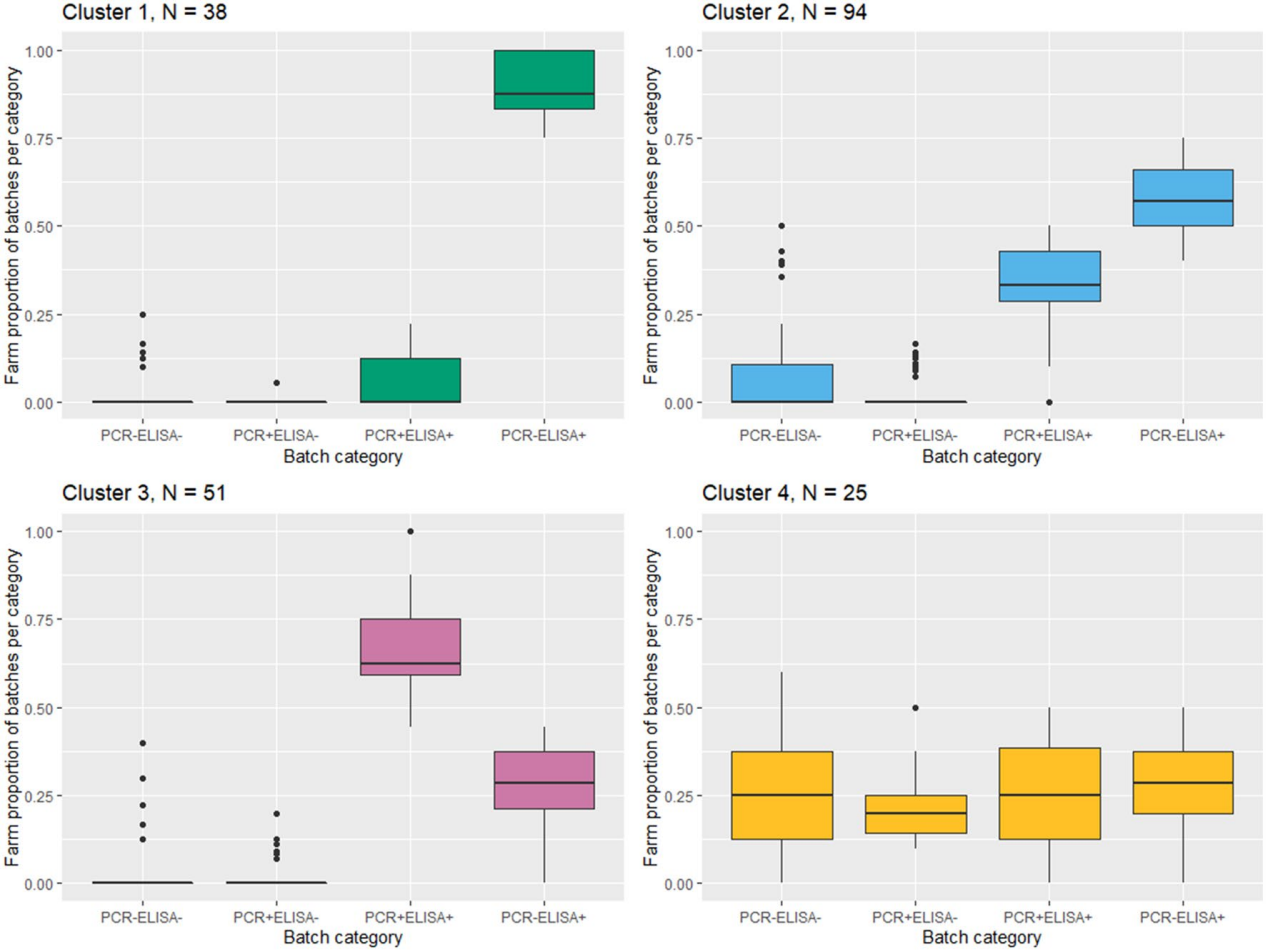
**Boxplots of batch category proportion values per farm, for four farm clusters.** Boxes represent the middle 50% of the values, marked with three horizontal lines that represent the first quartile (bottom), the median (middle) and third quartile (upper line).

The optimal number of clusters for the k-means clustering was 4, according to 8 out of 23 methods (Figure 3). Figure 4 shows for each cluster the median and 25^th^ and 75^th^ percentile for the batch categories PCR^−^ELISA^−^, PCR^+^ELISA^−^, PCR^+^ELISA^+^ and PCR^−^ELISA^+^ showing four different patterns. Farms of clusters 1 to 3 have a median proportion for PCR^−^ELISA^−^ and PCR^+^ELISA^−^ batches of 0. Farms of cluster 1 (number (*N*) = 38) delivered almost every batch to slaughter with a high seropositive proportion and without viraemic pigs (median for PCR^−^ELISA^+^ batch proportion is 0.9), suggesting relatively early age of HEV infection in these farms. Farms of cluster 2 (*N* = 94) and 3 (*N* = 51) differ especially in the ratio of PCR^+^ELISA^+^ and PCR^−^ELISA^+^ batches, with a median proportion for PCR^−^ELISA^+^ batches of 0.4 in cluster 2 and 0.7 in cluster 3. The smallest cluster 4 (*N* = 25) contains farms with no consistent infection pattern between batches, as every batch category is represented. Yet, 19 out of 25 farms have at least one PCR^−^ELISA^−^ batch, and all farms have a proportion of 0.1 to 0.5 PCR^+^ELISA^−^ batches, showing low transmission of HEV in some batches and late infections in other batches.

Almost all farm types are represented in each of the clusters (Table 4). About half of the sampled conventional farms are assigned to cluster 2 and a low percentage to clusters 1 and 4. The majority of organic farms (85%) are assigned to cluster 1, but none to cluster 3 and 4, and 80% of HyCare farms are assigned to cluster 2. High health farms are found in cluster 4 with late HEV infection and low transmission in some batches relatively often, compared to the other farm types (31%). The between sum of squares/total sum of squares of the four clusters was 0.78, meaning that 78% of the total variation was explained by variation between the clusters.

**Table 4:** **Summary statistics of proportion of farms, per farm type, and within-farm proportions of 4 batch categories, in 4 clusters determined by k-means clustering**

Cluster	Cluster 1	Cluster 2	Cluster 3	Cluster 4
Proportion of farms (N)	0.18 (38)	0.45 (94)	0.25 (51)	0.12 (25)
Proportion of organic farms (N)	0.85 (17)	0.15 (3)	0.00 (0)	0.00 (0)
Proportion of conventional farms (N)	0.12 (19)	0.48 (78)	0.28 (46)	0.12 (19)
Proportion of high health farms (N)	0.13 (2)	0.31 (5)	0.25 (4)	0.31 (5)
Proportion of HyCare farms (N)	0.00 (0)	0.80 (8)	0.10 (1)	0.10 (1)
Mean proportion of PCR^−^ELISA^−^ batches (IQR)	0.03 (0.0–0.0)	0.06 (0.0–0.11)	0.03 (0.0–0.0)	0.25 (0.13–0.38)
Mean proportion of PCR^+^ELISA^−^ batches (IQR)	0.00 (0.0–0.0)	0.02 (0.0–0.0)	0.02 (0.0–0.0)	0.22 (0.14–0.25)
Mean proportion of PCR^+^ELISA^+^ batches (IQR)	0.07 (0.0–0.12)	0.34 (0.29–0.43)	0.67 (0.59–0.75)	0.26 (0.13–0.38)
Mean proportion of PCR^−^ELISA^+^ batches (IQR)	0.90 (0.83–1.0)	0.58 (0.5–0.67)	0.28 (0.21–0.38)	0.27 (0.2–0.38)
Within cluster sum of squares	0.57	2.02	1.57	1.20

## Discussion

In the present study, selected farms were repeatedly sampled at slaughter and their pigs tested for HEV antibodies and RNA in serum to study variation in HEV population dynamics on pig farms. Four clusters of farms were identified that have different patterns of PCR and ELISA results among batches of pigs, reflecting differences in the relative age of HEV infection across the pig farms.

Clusters 1 to 3 contain farms that delivered almost exclusively HEV ELISA^+^ batches to slaughter. Cluster 1 farms have a median of 0.9 PCR^−^ELISA^+^ batches, meaning early age of HEV transmission and a high (indirect) contact rate in almost every batch, so presumably in every farm compartment. Farms in clusters 2 and 3 delivered PCR^+^ELISA^+^ and PCR^−^ELISA^+^ batches to slaughter. Because the batches of these farms are almost all ELISA positive, HEV infections likely occur in every batch. The ratio of PCR^+^ and PCR^−^ batches suggests different age of onset of transmission among farm compartments. Various studies suggest that mingling of pigs between the weaning and fattening phase at 10 weeks of age, results in a peak in HEV infected pigs at about 12 weeks of age [7, 15, 35]. However, HEV viraemia only lasts 1–2 weeks [36], so infection at 12 weeks of age would not result in PCR^+^ batches at slaughter (22–25 weeks of age). Therefore, farms in cluster 2 and 3 may have HEV transmission in PCR^−^ELISA^+^ batches due to mingling, but in the PCR^+^ELISA^+^ batches a later moment of HEV introduction may have occurred that cannot be linked to the moment of mingling around 10 weeks of age.

Cluster 4 contains farms that are able to deliver PCR^−^ELISA^−^ batches and PCR^+^ELISA^−^ batches to slaughter. This indicates that although HEV is present on the farms, HEV transmission is low in some batches, and is sometimes only introduced in a late stage of the fattening period. The farms do not resemble each other with regard to batch results and no distinct pattern can be elicited in the cluster. Other HEV cross-sectional farm seroprevalence studies reported farms being supposedly free from HEV (e.g. [14, 37, 38]). The few farms that were found HEV free in those studies, may have HEV batch infection patterns like the farms in cluster 4 and deliver certain batches HEV negative to slaughter.

Farm clustering partly coincided with the farm types that were included in this study. Organic farms were primarily distributed in cluster 1. We found that the odds of organic farms delivering an HEV PCR positive batch to slaughter are about ten times lower than the odds of conventional farms. In line with previous studies, we also found a high seropositive proportion of above 80% in organic farms [14, 18]. In the authors’ experience, organic farms are less compartmentalized than conventional farms and have closed floors covered with bedding materials where faeces can maintain for a longer period than on slatted floors. Both likely facilitate transmission of and exposure to HEV. This supports the idea that farms in cluster 1 have fast transmission of HEV early in the fattening phase or perhaps already before the start of the fattening phase, due to certain farm characteristics.

Maintaining batches free from HEV may be the result of proper internal biosecurity, i.e. preventing exposure to HEV from the environment as mode of introduction in a new batch and preventing transmission within the batch. However, the HyCare farms that were included in the study for having a better than usual internal biosecurity, did have a lower seropositive proportion than other farm types, but are not represented by cluster 4 with PCR^−^ELISA^−^ batches. In hindsight, some included HyCare farms were either starting or ending with the HyCare concept©. Therefore, the HyCare farms included in the study may not have an internal biosecurity level as expected and that may explain their allocation in other clusters than cluster 4. High health farms, that were mostly SPF farms or farms receiving pigs from SPF farms, had similar farm seropositive and PCR positive proportions as conventional farms. Still, although only based on twenty farms, a third of high health farms belonged to cluster 4, suggesting that these farms may be better able to keep batches free from HEV infection.

Selecting farms of specific types has the advantage that sufficient farms are included to investigate that type, but the disadvantage that it violates random selection. The inclusion criterion to have at least ten deliveries of pigs to slaughter per year also violates random selection and may have caused selection bias towards larger pig farms and or continuous pig flow. Nevertheless, without that inclusion criterion, there could have been a higher loss to follow-up and more farms with too few batches to determine the variation. Because almost every batch that was delivered to slaughter was sampled, the number of batches per farm varied. This does not matter for the estimations of farm-level seropositive and PCR positive proportions, but may have affected the clustering of farms slightly, because 1/4 PCR^−^ELISA^−^ batches is equal to 3/12 PCR^−^ELISA^−^ batches in the k-means clustering method, whereas 3/12 results in more certainty about the ability of a farm to deliver HEV free batches to slaughter. These limitations must be considered when evaluating the outcomes.

The low sample size on batch level of on average six samples is another limitation in the study. A batch of slaughter pigs usually includes around 212 pigs, so there is a high probability of missing infections in case of a low number of infected pigs. Yet, a low seroprevalence is not likely, because HEV in pigs has an estimated reproduction number of 5 to 9, giving a probability of a large outbreak in homogenously mixed populations of 80–89% [39, 40]. Viraemic pigs may have been missed because of the batch sample size. However, the estimated 10% of viraemic pigs (Additional file 2) batches is comparable with results of other studies, for instance to the 15% of Dutch pigs at slaughter found positive in faeces before [41]. Thus, despite a low batch level sample size, both ELISA and PCR results seem accountable.

This study focused on HEV in batches of slaughter pigs. HEV PCR^−^ batches are not necessarily batches without actively infected pigs at slaughter. A PCR^−^ batch demonstrates that the sampled pigs in the batch do not have a viremia, yet faecal shedding of HEV lasts longer than viremia (up to 7 weeks) [39, 42] and infection of the liver and other organs may outlast shedding [43]. HEV ELISA^−^ batches may contain 1/6 seropositive pigs. A batch at slaughter is not always equal to a compartment of pigs on a farm. Pig farmers are primarily focused on delivering pigs of comparable and desired weight and fat composition. Consequently, farmers may select pigs from different compartments and ages to be combined into one slaughter batch. This combination of pigs from different compartments may have increased the number of ELISA^+^ batches, as a batch is already considered ELISA^+^ when 2/6 pigs are seropositive. In line with the fact that batches may not perfectly resemble farm compartments, accepting one seropositive pig in ELISA^−^ batches seems justified.

All in all, this study demonstrates that farms have distinct patterns of batch HEV infection dynamics. The relative age of HEV infections varies between farms, but for some farm clusters also within farms. The variation partly coincides with farm type, presumably because of differences in biosecurity and ways of transmission of HEV across farm compartments. Repeated cross-sectional sampling of batches at slaughter has added value in eliciting infection dynamics of HEV on farms in a cost-effective way without requiring sampling of live pigs. However, longitudinal studies on farms from specific clusters are needed to uncover the relation between population infection dynamics and mitigation measures. Follow-up of farms that are able to keep some compartments free from HEV despite presence of HEV in the farm may reveal practices to reduce HEV transmission within farms. This study provides new insights and implies the need for future risk factor and mitigation strategy studies for HEV within farms.

## Supplementary Information

**Additional file 1: Comparison of the performance of two ELISAs with different ways to purify antigen.** Summary of the analysis to determine the cut-off of an adjusted antibody ELISA with the same antigen coating compared to the original ELISA but a different way of purifying the antigen and a lower dilution of coating material used, and of a difference in the seroprevalence estimates of 87 farms dependent on the ELISA used.

**Additional file 2: Estimation of the average percentage of viraemic pigs in slaughter batches.** Summary of the analysis to estimate the percentage of viraemic pigs in a batch, given that the pooled batch results of on average 6 pigs is PCR positive.

## Data Availability

The datasets generated and/or analysed during the current study are available in the Yoda repository, DOI: 10.24416/UU01-FME49I (10.24416/UU01-FME49I).

## Abbreviations

AIC: Akaike’s information criterium
CI: confidence interval
ELISA: enzyme-linked immunosorbent assay
ELISA^−^ / ELISA^+^: a batch of on average 6 pigs at slaughter with either 0 or 1 seropositive pigs (−) or 2 or more ( +)
HEV: hepatitis E virus
HRPO: horse radish peroxidase
IQR: interquartile range
N: number
OD: optical density
OR: odds ratio
PCR: polymerase chain reaction
PCR^−^ / PCR^+^: a pooled PCR result of a batch of on average 6 pigs at slaughter that is either negative (−) or positive ( +)
PP: percentage positive
RNA: ribonucleic acid
SPF: specific pathogen free
TMB: 3,3',5,5'-Tetramethylbenzidine
