# Supplementary material for: Repeated cross-sectional sampling of pigs at slaughter indicates varying age of hepatitis E virus infection within and between pig farms
Source: Vet Res. 2022 Jul 7;53:50. doi: 10.1186/s13567-022-01068-3 (PMC9264715; doi:10.1186/s13567-022-01068-3)
Supplement: Supplementary file 1 — Additional file 1: Comparison of the performance of two ELISAs with different ways to purify antigen. Summary of the analysis to determine the cut-off of an adjusted antibody ELISA with the same antigen coating compared to the original ELISA but a different way of purifying the antigen and a lower dilution of coating material used, and of a difference in the seroprevalence estimates of 87 farms dependent on the ELISA used. [file 13567_2022_1068_MOESM1_ESM.docx]

## Additional file 1 Comparison of the performance of two ELISAs with different ways to purify antigen

Serum of 11 115 pigs at slaughter was collected to test for HEV antibodies. All sera were tested with an in-house pig specific IgM and IgG ELISA. However, due to discontinuation of the manufacturer of the original antigen coating in the ELISA, the antigen coating was retrieved with a new purification method and was used to test a part of the samples. As a result, sera collected between January and June 2019 were tested with the originally coated ELISA, *N* = 7984, and sera collected between June and August 2019 were tested with the alternatively coated ELISA, *N* = 3131.

The cut-off of the originally coated ELISA is 24.5 PP. The cut-off of the alternatively coated ELISA was determined by analysing 800 sera with both the original and alternative ELISA, and creating an ROC curve, calculating at what cut-off the highest percentage of sera had equal outcomes with both tests and with Cohen’s kappa. By combing the results of these three analyses, the cut-off was set at 43.5 PP.

To assess whether the change in coating influenced the results of the study, results of both ELISAs with the above given cut-offs were compared on within-farm results, for all farms that had at least two batches tested with the originally and with the alternatively coated ELISA (*N* of farms = 87).

The average seropositive proportion was calculated per ELISA test per farm and the difference between the two proportions was calculated. Figure A1 shows a histogram of the differences between both seropositive proportions. For more than 35 out of 87 farms the difference was less than 0.1. The differences are normally distributed around zero (Shapiro-Wilk normality test: W is 0.99, *p*-value is 0.49).

A one sample T-test of the differences was done, to test whether the difference between the ELISAs is 0 or not. The observed mean difference is – 0.0045, with a *p*-value of 0.84 and a 95% confidence interval of -0.05 – 0.04. Therefore, it can be concluded that the change in coating did not systematically affect the within-farm seropositive results in the study.


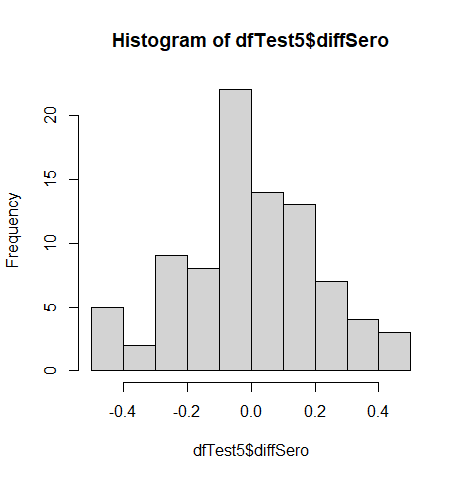


**Figure A1 Histogram of the difference between within-farm HEV seropositive proportion according to originally and alternatively coated ELISA**
