# Supplementary material for: Repeated cross-sectional sampling of pigs at slaughter indicates varying age of hepatitis E virus infection within and between pig farms
Source: Vet Res. 2022 Jul 7;53:50. doi: 10.1186/s13567-022-01068-3 (PMC9264715; doi:10.1186/s13567-022-01068-3)
Supplement: Supplementary file 2 — Additional file 2: Estimation of the average percentage of viraemic pigs in slaughter batches. Summary of the analysis to estimate the percentage of viraemic pigs in a batch, given that the pooled batch results of on average 6 pigs is PCR positive. [file 13567_2022_1068_MOESM2_ESM.docx]

Serum samples per batch of slaughter pigs are pooled and tested by PCR for HEV RNA presence. The outcome per pool is “negative” (no RNA) or “positive” (RNA present). The probability that pool *i* of farm *j* tests negative, *P_ij_*(0), is dependent on the prevalence of viraemic pigs on farm *j*, π*_j_*, and the number of samples in that pool, *n_ii_*, according to:

$$P_{ij}\left( 0 \right)=\left( 1-\pi_{j} \right)^{n_{ij}}$$

The probability that a pool tests positive, because serum of at least one viraemic animal is included, equals:

$$P_{ij}\left( 1 \right)=1-P_{ij}\left( 0 \right)=1-\left( 1-\pi_{j} \right)^{n_{ij}}$$

The subsequent likelihood function for *n_i_* batches of *n_j_* farms, with *x_ij_* representing the PCR results, equals:

$$L\left( \pi_{j} | x_{ij} \right)=\prod_{j=1}^{n_{j}} \prod_{i=1}^{n_{i}} 1-\left( 1-\pi_{j} \right)^{n_{ij}}$$

Analyses were done in R version 3.5.1, using the package rJAGS [44]. π*_j_* were considered to be beta-distributed with parameters α=πϕ and β=(1− π)ϕ, with π the average prevalence of viraemic animals and ϕ the precision. Parameter estimates were obtained as posterior distributions by Markov Chain Monte Carlo sampling. Five Markov chains were ran simultaneously with randomly chosen initial values. The first 1000 iterations were discarded for burn-in and no thinning was applied. Priors for π and ϕ were specified as vague: β(1,1) for π and γ(0.001, 0.001) for ϕ. Priors were changed in a sensitivity analysis to assess their influence.

In total, 1711 PCR results from 1711 batches of 208 farms were included in the analysis. Of these, 679 tested positive (40%). The average prevalence of viraemic animals among these farms was estimated at 9.6% (95% credibility interval: 8.7–10.6%) (Figure A2). The use of alternative priors did not affect these outcomes, as the differences were within the range of de MCMC sampling variability.


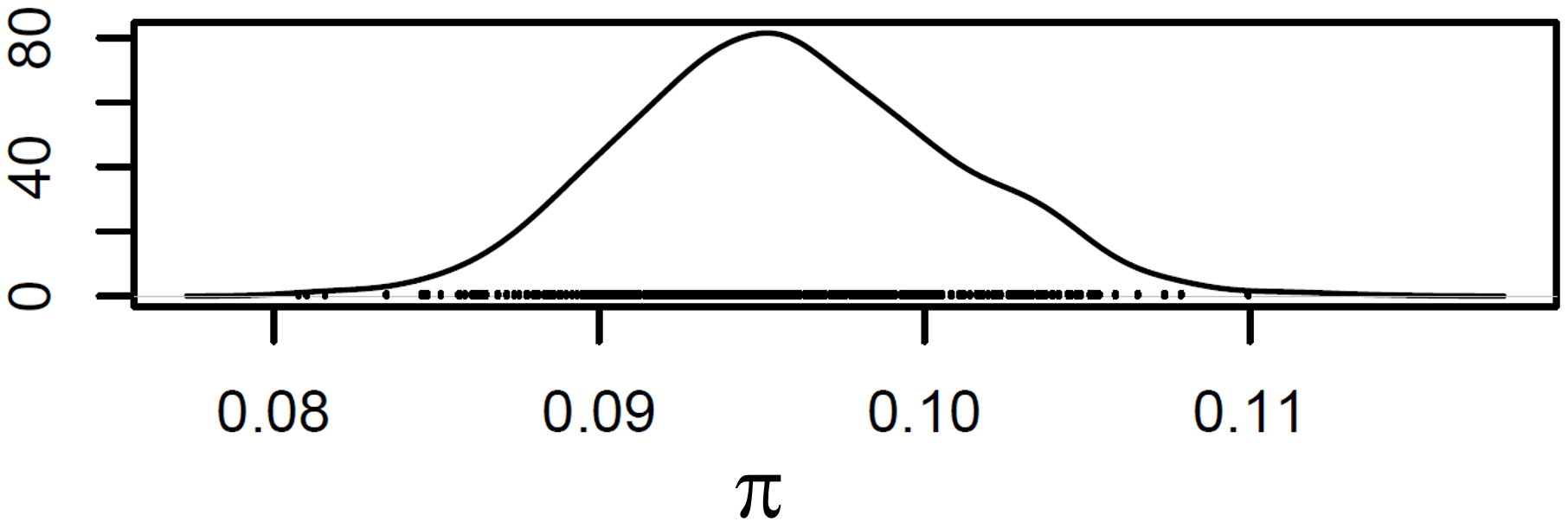


**Figure A2 Posterior distribution of the average percentage of viraemic pigs per farm.**
